# Preparing the way for novel blood-based biomarkers and disease-modifying treatments for Alzheimer’s disease in the NHS in the UK

**DOI:** 10.1177/01410768241311139

**Published:** 2025-01-28

**Authors:** Roxanna Korologou-Linden, Lefkos Middleton, Azeem Majeed, Dasha Nicholls, Oliver Robinson, Benedict Hayhoe

**Affiliations:** 1 Ageing Epidemiology (AGE) Research Unit, School of Public Health, Imperial College London, London W6 8RP, UK; 2 NIHR Applied Research Collaboration Northwest London, London W12 0BZ, UK; 3 Department of Primary Care and Public Health, Imperial College London, London W12 0BZ, UK; 4 MRC Centre for Environment and Health, Department of Epidemiology and Biostatistics, School of Public Health, Imperial College London, London W12 0BZ, UK; *Contributed equally.

Dementia has been the UK’s leading cause of mortality over the last decade, affecting nearly one million individuals, with Alzheimer's disease (AD) representing the majority.^1^ AD is characterised by the presence of amyloid (A) plaques, aggregation of intraneuronal tau (T) tangles and neurodegeneration (N) (ATN signature).^2^ AD for many individuals has a long preclinical phase, with asymptomatic ATN changes developing over up to two decades,^2^ yet most AD is identified only in the late symptomatic phase.

This is expected to change dramatically with the advent both of blood-based biomarkers and novel disease-modifying therapies (DMTs), which hold promise for transforming AD identification and care. We explore how the National Health Service (NHS) in the UK is equipped to adopt these innovations and integrate them into its care model.

## What pharmaceutical agents are available for Alzheimer’s disease?

National Institute for Health and Care Excellence (NICE) guidance recommends acetylcholinesterase inhibitors (AChEIs) for the management of mild to moderate AD, and the addition of memantine alone or with AChEIs to manage moderate/severe AD.^3^ However, while these may result in small improvements in cognitive and behavioural symptoms, they do not modify disease progression.

After two decades of unsuccessful pharmaceutical research, there have recently been breakthroughs in anti-amyloid DMTs of monoclonal antibody (MAB) therapies in early symptomatic AD.^4^ Two US-approved anti-amyloid MAB therapies, lecanemab (also approved in the UK (excluding Northern Ireland) from August 2024) and donanemab, significantly reduce brain amyloid load and cognitive decline, on average by four to six months, if administered early in the disease. MABs are currently being tested in cognitively unimpaired individuals at high risk, as there may be potential for preventative targeting of the large ‘preclinical AD’ population.^5,6^ However, MABs are costed at a high price and present risks such as amyloid related imaging abnormalities (ARIA) in individuals homozygous for the *APOE* ε4 allele. Hence, these therapies require reliable evidence of the ATN signature and cannot be administered based on clinical diagnosis alone.

## Does the current pathway to diagnosis stand in the way of effective therapies?

A definitive diagnosis of AD is based on the ATN pathological signature, confirmed at autopsy. The next best *in vivo* diagnosis relies on identification of pathological markers through positron emission tomography (PET) or cerebrospinal fluid (CSF) sampling. However, these tests are costly and invasive (approximately £1350 per PET scan and £700 per CSF test in 2023 currency^7^), and until recently, there has been little incentive to perform them due to the lack of effective DMTs. This has resulted in the absence of a clear, universal diagnostic framework for AD across disease stages, with only an estimated 2% of individuals in the UK being diagnosed with AD using these methods.^8^

Currently, the diagnostic pathway for AD is based on clinical symptoms emerging late in the disease, often after 20 years of progressive accumulation of intracerebral pathological AD features. When memory, mood or personality changes are noticed, individuals or their family usually seek advice from a general practitioner, who may perform basic validated cognitive testing. If cognitive decline is suspected, the patient is referred to a memory clinic, typically old Age Psychiatry Clinics within Mental Health Trusts, for further evaluation, aimed at confirming cognitive decline and ruling out reversible causes. NICE guidelines recommend fluorodeoxyglucose (FDG) imaging or CSF testing when AD is suspected or when subtyping dementia would be beneficial.^9^

However, access to memory clinics is limited by complex pre-referral criteria and long wait times.^10^ Only 64% of individuals over 65 years of age estimated to have dementia are formally diagnosed in primary care, which affects epidemiology and health planning.^11^ In England and Wales, the National Audit of Dementia shows the average wait time from referral to diagnosis has increased to 17.7 weeks from 13 weeks in 2019, with this ranging from 0 to 2 years nationwide.^12^

Despite the recent licensing of lecanemab as the first ever AD-modifying drug approved in the UK (excluding Northern Ireland) by the Medicines and Healthcare Products Regulatory Agency (MHRA) for individuals with early AD (except for *APOE* ε4 allele homozygotes), the drug’s appraisal by NICE was unfavourable.^13^ This decision was attributed to the cost of providing the drug, including fortnightly drug infusions and intensive monitoring for side-effects in combination with the relatively small cognitive benefits. However, economic modelling included the costs associated with diagnostic testing for amyloid pathology in individuals with AD who would not otherwise have been tested.^14^ Given a very small proportion of individuals diagnosed with AD receive confirmatory testing with CSF and PET tests due to high cost/inaccessibility, the current approach to diagnosing AD within the NHS likely dramatically affected the output of such economic modelling.

Dementia diagnostic and imaging costs are estimated at £80 million annually, just 0.2% of total spend on dementia care, yet 63% of the financial burden falls on patients and their families. As disease severity increases, the annual cost per person rises significantly (mild: £28,700; moderate: £42,900; severe: £80,500).^15^ With DMTs now available, early diagnosis could lead to significant cost savings by preventing disease progression. However, unlike conditions like stroke, where clinical pathway changes (e.g. centralisation of hyperacute stroke units and integration of care pathways) led to immediate benefits, the impact of AD interventions may only be seen over decades to come.^16^

## Is the NHS ready for these drugs?

Prior to the UK regulatory approval of lecanemab, report titles^17^ such as the ‘NHS “not ready” for Alzheimer’s wonder drugs as patients face postcode lottery for brain scans’ warned of location-based inequalities in access to the new treatments. Licensing in the UK by MHRA with lack of NICE approval for NHS prescribing means this inequality in access is now a reality. The negative recommendation highlights NHS shortcomings in terms of capacity to identify eligible individuals, administer treatment and monitor adverse effects.

Alzheimer’s Research UK and the Royal College of Psychiatrists investigated the diagnostic and service challenges encountered by old-age psychiatrists. They found that only 36% of psychiatrists believed their services could adapt to deliver DMTs within a year and only 6% of psychiatry services fully comply with NICE guidelines in accessing additional biomarker and diagnostic tests for AD.^16^ There are prerequisites such as the integration of genetic testing into the diagnostic pathway, due to a higher prevalence of ARIA following anti-amyloid MAB administration in homozygous *APOE* ε4 allele carriers.^4^ DMTs also necessitate regular drug administration and monitoring for side-effects. While amyloid PET scans serve as a crucial diagnostic tool for AD and are essential for accurately assessing treatments’ impact on reducing brain amyloid burden, they are not currently recommended by regulatory bodies in the UK and globally due to insufficient evidence of clinical utility and cost-effectiveness.^4^

Currently, the NHS is not set up for MABS due to the current diagnostic tests being inaccessible, expensive or invasive and the lack of infrastructure for administration and monitoring of drug effects. There is an urgent need for accessible, cost-effective diagnostic tools for primary care, where most memory service referrals originate. Once effective early diagnosis is achieved, streamlining the AD clinical pathway and fostering collaboration among neurology, geriatrics and radiology is essential to improve patient access to specialists.^16^

## Are blood-based biomarkers for Alzheimer’s disease ready for implementation in NHS primary care?

AD has evolved from being defined purely by clinical symptoms to a biological construct, due to technological advances in biomarker identification such as CSF and PET methods. Over the last decade, mass spectrometry-based and fully automated immunoassay methods measuring blood-based biomarkers have shown similar sensitivity and specificity to PET and CSF tests in clinical research settings.^18^ If reliably predictive blood tests were to become available at low cost in primary care, this could be transformative for AD identification^4^: One US study found that combining cognitive and blood-based biomarker tests could eliminate wait lists in three years, halve costs and improve AD diagnostic accuracy.^19^ However, blood-based biomarkers have not yet been established as a standalone diagnostic tool, mainly due to a gap in evidence validating their comparability to CSF and PET imaging in real-world scenarios. Initiatives like the Davos Alzheimer’s Collaborative (DAC) International Initiative, the AD RIDDLE study^20^ and the UK’s ‘Blood Biomarker Challenge’^21^ aim to pilot real-world implementation and validation of blood-based biomarkers, with hopes of integrating them into the NHS within the next few years.

## What next?

The negative decision regarding the use of lecanemab in the UK highlights the ongoing challenges in delivering effective Alzheimer’s treatments. However, the rationale of this decision also sparks hope for new therapies in the pipeline. Trontinemab, for example, is an innovative drug that uses brain shuttle technology to cross the blood–brain barrier, potentially offering improved efficacy with fewer side-effects. Alongside these advancements, clear guidelines for diagnosis and treatment, taking into account new developments, are essential. Innovations like blood-based biomarkers and dried blood spot testing promise to revolutionise early detection, making Alzheimer’s diagnosis more accessible, especially for people in remote areas. For successful introduction into the NHS, novel investigations and treatments must undergo regulatory approval by the MHRA and cost-effectiveness evaluation by NICE, with clear pathways for reimbursement. Collaborative efforts between healthcare providers, Alzheimer’s charities and policymakers will be key to integrating these treatments into memory services and primary care. Clear guidelines must also be developed to ensure that caregivers are supported, and resources are culturally inclusive. With a focus on affordability, accessibility and robust post-launch monitoring, an Alzheimer’s care framework that is more effective, equitable and capable of transforming the lives of those affected by the disease can be created.

## Declarations

### Guarantor

RKL and BH.

### Provenance

Not commissioned; peer-reviewed by Nishant K. Mishra.
